# Obtaining new cultures of microorganisms that produces cellulases and xylanases from the sugarcane bagasse

**DOI:** 10.1186/1753-6561-8-S4-P102

**Published:** 2014-10-01

**Authors:** Ludmylla Noleto, Daniella Moreira, Fabrícia Faria

**Affiliations:** 1 Universidade Federal de Goiás, Brazil

## Background

The international energetic system strongly depends on fossil fuels, which causes negative effects in the environment, such as the global warming. Biofuels appear as an environmental and economic alternative for the energetic industry because of their potential source of renewable energy. Several studies are based on sugarcane culture and its derivatives, as bagasse, the sugarcane residue. Bioethanol can be produced by the fermentation of sugar or by the hydrolysis of cellulosic biomass [1]. The plant cell wall is constituted of cellulose (40-50%), hemicellulose (15-30%) and lignin (10-30%), forming the vegetal biomass. Cellulases are enzymes that form a complex that hydrolyses cellulosic materials, releasing sugars [2]. The main component of hemicellulose is the xylan, which is hydrolyzed by xylanases [3]. Cellulases as xylanases have a great biotechnological potential, they can be used in a variety of field: food, animal feed, textile and paper recycling industries. The sugarcane bagasse (SCB) is the most studied lignocellulosic waste for bioethanol production, because it is a by-product of conventional ethanol and can be find in large amount in Brazil [4]. Nowadays, the process of bioconversion of biomass has high cost and low specific activity of the enzymes that are necessary for the cellulose saccharification [5]. The aim of this research is to obtain microorganisms that hydrolyze the sugarcane bagasse and to quantify the sugar production.

## Methods

The microorganisms present in the SCB were isolated from three preparations: fresh SCB, SCB buried in soil for about 45 days and humid SCB - collected from two cane fields and stored in refrigerator. To obtain microorganisms, saline solution (NaCl 0,15 M) and rich medium (5 g/L peptone, 5 g/L NaCl and 10 g/L of SCB, pH 5.0 to 6.0) were used, followed by serial dilution. The selection medium contained cellulose and xylan and the enzymatic activity was visualized as a halo of hydrolysis around the culture, using congo red 1%. Submerged fermentation in minimum medium (MM) was used to induce cellulases and xylanases. The determination of enzymatic activity was measured by dinitrosalicilic acid (DNS), using the supernatants of culture as enzymes and Xylan *birchwood*-Sigma^®^, CMC-Sigma^®^, Avicel-Sigma^® ^and Whatman paper filter as substrate for each enzymatic dosage.

## Results and conclusions

Seven cultures were selected (A3, B3, M2, M3, X7, F4 and D2) according to the halo of hydrolysis diameter to determine the enzymatic activity. The culture A3 proved to be a good producer of xylanase. The culture M3 produced cellulases with FPase and CMCase activity, showing that is good for cellulose hydrolysis. The culture X7 simultaneously produces cellulases and xylanases, which favors the hydrolysis of cellulose and hemicellulose using SCB as substrate. Although the activity of avicelase has no results, we cannot conclude that the enzyme was not produced; the microorganisms need to be induced by different specifications.
